# Rare presentations of primary amyloidosis as ptosis: a case report

**DOI:** 10.1186/s12886-022-02267-4

**Published:** 2022-01-29

**Authors:** Peng-Hsuan Lee, I-Chuang Liao, Wan-Ju Annabelle Lee

**Affiliations:** 1 Department of Ophthalmology, National Cheng Kung University Hospital, College of Medicine, National Cheng Kung University, Tainan, 704 Taiwan; 2 Department of Pathology, Chi Mei Medical Center, Tainan, 710 Taiwan; 3 Department of Ophthalmology, Chi Mei Medical Center, Tainan, 710 Taiwan; 4 School of Pharmacy & Institute of Clinical Pharmacy and Pharmaceutical Sciences, College of Medicine, National Cheng Kung University, 701 Tainan, Taiwan; 5 Department of Optometry, Chung Hwa University of Medical Technology, Tainan, 717 Taiwan

## Abstract

**Background:**

Amyloidosis is a rare, progressive and variable group of diseases characterized by extracellular deposits of amyloid protein in different tissues and organs. It is a protein-misfolding disease in which small proteins of about 10 to 15 kDa acquire an alternative and relatively misfolded state at minimum energy and subsequently aggregate into oligomers and polymers. It mimics other eyelid diseases, such as involutional ptosis, eyelid granulomatous or cancerous lesions. Misdiagnosis of eyelid amyloidosis is usual when the lesion grows slowly and insidiously. Definite diagnosis depends on clinical suspicion and tissue-proven biopsy.

**Case presentation:**

A 50-year-old female had painless progressive ptosis in both eyes for 6 months. She presented with limited upward gaze due to swelling of the upper eyelids OU. She complained of mild foreign body sensation. Upon examination, we observed an infiltrated irregular yellowish mass on the surface of her upper palpebral conjunctiva in both eyes. The mass was non-movable without tenderness. We performed excisional biopsy for the masses and subsequent histopathology of the biopsy specimens revealed amyloidosis. Systemic workup showed no other lesions. Unfortunately, her ptosis and upward gaze restriction was not improved after the operation. However, the masses did not enlarge in the following 3 months.

**Conclusions:**

The varied presentations of ocular adnexal and orbital amyloidosis often lead to a significant delay between first symptoms and diagnosis. Immediate confirmatory biopsy and subsequent systemic workup should be performed whenever amyloidosis is highly suspected.

**Keywords:**

Amyloidosis Conjunctival mass Ptosis

## Background

Amyloidosis is classified based on the precursor protein that forms the amyloid fibrils and the distribution of amyloid deposition (localized or systemic). It is classified either as local, in which the amyloid is restricted to one particular organ or tissue type [1]; or systemic, in which the amyloid deposition occurs in two or more distinct sites [2]. Ocular adnexal and orbital amyloidosis is a rare presentation and can occur with localized or systemic disease [3–11]. Due to its rare occurrence, only case reports or case series have ever been reported. The importance of systemic survey for orbital or ocular adnexa amyloidosis lies in the fact that different types of ocular presentation can be brought forth, along with their incidence and association with systemic amyloidosis [12]. On rare occasion ocular amyloidosis has been reported in association with some malignancies, such as multiple myeloma, extranodal marginal zone lymphoma, and plasma cell myeloma [13]. The gold standard diagnostic procedure for amyloidosis is histopathological biopsy examination [2], with apple-green birefringence under Congo-red stain.

## Case presentation

A 50-year-old female presented with progressive, painless ptosis with limited upward gaze in both eyes. She had foreign body sensation OU very often, especially when she blinked. There was no personal or family history of systemic disease, including multiple myeloma or any neurologic or autoimmune diseases. The patient had never undergone dialysis.

**Fig. 1:**
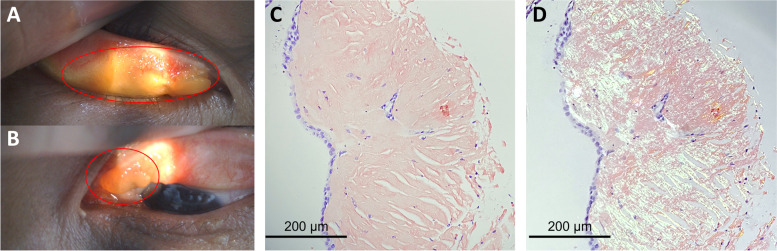
**A** & **B**: Slit lamp photograph showing yellowish calcified plaques of both eyelids (*red circles*) (A: right eye; B: left eye). **C**: Section reveals positive Congo red staining in the stroma (salmon color). (original magnification X400). **D**: When viewed under polarized light, apple green birefringence was identified and confirmed the diagnosis of amyloidosis. (original magnification X400)

**Fig. 2:**
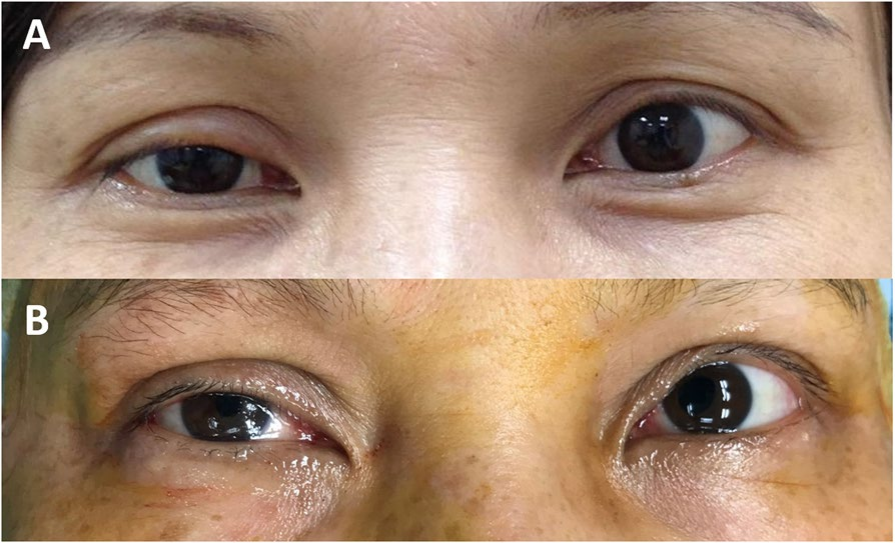
**A**: Pre-operative photograph revealing ptosis of both eyes (MRD1 = 1 mm OD, MRD1 = 3 mm OS). **B**: Post-operative photograph revealing ptosis of both eyes (MRD1 = 1 mm OD, MRD1 = 3 mm OS)

On examination, her visual acuity was 20/20 bilaterally. Pupils, intraocular pressure, extraocular motility, and alignment were within normal limits. The margin reflex distance 1 (MRD1) was 1 mm OD and 3 mm OS. Her palpebral fissures were 6 mm OD and 8 mm OS. Levator function was normal and symmetrical bilaterally. Slit lamp examination revealed yellowish calcified plaques in her bilateral upper palpebral conjunctiva (Fig. 1A and B, *red circle*s), which tended to bleed easily when we everted the upper eyelids. Eversion of the other 2 eyelids (lower eyelids) showed no abnormalities. The remainder of the ocular examination was unremarkable. We subsequently performed excisional biopsies for the yellowish masses in both eyes. Histopathological examination revealed eosinophilic, hyaline extracellular material which, when stained with Congo red (Fig. 1C) produced apple-green birefringence under polarized light (Fig. 1D). Formalin-fixed paraffin-embedded tissue specimens were prepared for hematoxylin-eosin and Congo red staining. The microscopic pictures were captured using Olympus BX53 microscope and cellSens software. Olympus U-POT Polarizer and U-ANT analyzer were used for polarization. Photoshop software was applied to adjust the brightness of polarized pictures. To rule out systemic amyloidosis, we referred the patient to the hematology department for further evaluation. The hematologist checked her complete blood count (CBC), metabolic panel, serum protein electrophoresis (SPEP), immunoelectrophoresis-serum (IEP-S), spot urine, and urine protein electrophoresis (UPEP), which were all within normal limits. Chest X-ray was negative for mass-like lesions. Abdominal sonography was performed with no evidence of amyloid deposition in the liver, kidney or spleen. Brain and orbital magnetic resonance imaging (MRI) was obtained which showed enhancing lesion isolated in the superior preseptal soft tissue at both eyes, in immediate proximity to our excision location. No intracranial deposits were observed in her image study. Finally, the patient was diagnosed as primary conjunctival amyloidosis without systemic involvement. Three months after the surgical removal the lesions of both eyes had not recurred. Her foreign body sensation improved dramatically, but her ptosis and upward gaze restriction did not improve after the operation. The post-operative MRD1 remained 1 mm OD and 3 mm OS. (Fig. 2A and B).

## Discussion and conclusions

We report a rare case of primary ocular amyloidosis without systemic involvement and confirmed by histopathological diagnosis. The case presented as ptosis, a common symptom in the aged population, which is easily overlooked if physicians do not check all parts of the eyes carefully. These deposits of proteins do not lead to vision disturbance or other intraocular lesions. Thus they are often misdiagnosed as other involutional eye diseases. In our case, the patient had consulted several other physicians before coming to our department. Most doctors diagnosed the case as involutional ptosis and gave her topical eye drops. The swelling of her eyelids and ocular discomfort persisted, which led us to evert her upper eyelids whereupon we discovered the calcified plaques. Her ptosis and upward gaze limitation was due to myogenic ptosis caused by the infiltrative amyloidosis, because when we performed the biopsy, the masses could not be excised totally.

Amyloidosis may involve any organ or system in the body. The heart, kidneys, gastrointestinal system, and nervous system are most often affected. Moreover, amyloid deposition may lead to organ failure or even death. Systemic amyloidosis with amyloid deposits in the viscera, blood vessel walls and connective tissue is usually fatal and is the cause of about one per thousand deaths in developed countries [2]. Reynolds et al. [12] reported a retrospective chart review, and found that 11.8% (8/68) of systemic amyloidosis patients had ophthalmic presentations. Kang et al. [13] investigated the natural history of ocular and orbital amyloidosis, and found that 10% of patients (4/41) had systemic amyloidosis, and 15% (6/41) had co-existing malignancies. Therefore, patients presenting with amyloid accumulations in one organ system should be worked up for systemic involvement. Lack of awareness leads to late detection and far advanced disease at presentation. Early diagnosis and early treatment are necessary to stabilize the disease and prevent disease progression.

Primary ocular amyloidosis is rare and often reported in case reports or a small scale case series [3, 6, 8–11, 13–23]. One such case series study discussed the histopathological and epidemiological pattern for 11 cases and concluded that most cases of primary ocular amyloidosis had no systemic amyloidosis and surgical removal was adequate treatment for primary ocular amyloidosis [24]. Another case series study by Kang et al. reviewed 41 cases of ocular amyloidosis and found that the most common symptoms were those of conjunctival mass (83%; 34/41) and ptosis (37%; 15/41), though the clinical condition presented in many different ways, such as a lacrimal gland mass with ptosis, recurrent tarsal masses and fibrosis or persistent caruncular mass [13]. One case report had been diagnosed as cutaneous amyloidosis based on the initial presentation with bilateral eyelid masses. The case had no systemic involvement although the masses were present all over the 4 eyelids and bilateral cheeks [25]. Sometimes, amyloidosis can cause corneal deposition and present as corneal opacities, which can mislead physicians into considering other common corneal diseases, such as drug-induced vortex keratopathy, Fabry disease, or malignancies like multiple myeloma and gammopathies [10]. One further case report presented as lymphoproliferative lesion in the beginning because of its presentation as a linear, slowly-growing lesion on the lower conjunctival fornix, which was revealed to be conjunctival amyloidosis after histopathological examination [11].

Most cases of localized ocular amyloidosis require surgical excision. Cui et al. [24] found their cases of primary ocular amyloidosis were not related to systemic diseases, and surgical removal of the lesion was adequate. However, other literature has pointed out that in addition to surgical excision, radiotherapy was required for lesions which could not be totally removed. Still observation was another treatment choice if no systemic involvement of the ocular amyloid was found [13]. In our case, after we had tissue-proved the amyloidosis, the patient underwent systemic survey that found no evidence of systemic disease. However, the calcified masses in the upper eyelids of both eyes could not be excised totally because they had infiltrated the underlying tissues, including levator aponeurosis and part of the superior rectus muscle. This explains why the post-operative orbital MRI showed enhancing lesion isolated to the superior preseptal soft tissue. We suggested the patient undergo radiotherapy for the residual lesions, but they refused after thorough physical examination. Thus, we suggested the patient to have regular ophthalmic follow-up every 3 months. And the hematologic specialist suggested regular follow-up every 6 months to do systemic workup.

The above review of the literature reinforces several important points. First, orbital amyloidosis presents with a wide variety of clinical signs, thus hindering its diagnosis. Only with a high degree of suspicion of this disease will physicians undertake the appropriate procedures to reach a final definite diagnosis. Second, although most ocular or adnexal amyloids are localized, systemic survey for other organ involvement is essential because systemic amyloid is related to high morbidity and mortality [2]. Detailed history examination and laboratory investigation must be implemented on suspicion of amyloidosis, because early recognition of systemic amyloidosis and appropriate treatment can improve clinical outcomes. Third, although surgical excision is the most common treatment, observation or radiotherapy have been used when there was no evidence of systemic involvement [16]. Lastly, long-term follow-up is required because amyloidosis has a substantial rate of recurrence.

## Data Availability

Not applicable.

## Abbreviations

OU: both eyes
OD: right eye
OS: left eye
MRD: margin reflex distance
CBC: complete blood count
SPEP: serum protein electrophoresis
IEP-S: immunoelectrophoresis-serum
UPEP: urine protein electrophoresis
MRI: Magnetic Resonance Imaging

## References

[1] Pepys MB (2006). Amyloidosis. Annu Rev Med.

[2] Hazenberg BP (2013). Amyloidosis: a clinical overview. Rheum Dis Clin N Am.

[3] Kaliki S, Morawala A, Gowrishankar S (2018). Primary eyelid amyloidosis presenting as a calcified plaque: a rare presentation. Ophthal Plast Reconstr Surg.

[4] Di Bari R, Guerriero S, Giancipoli G, Cantatore A, Sborgia G, Piscitelli D (2006). Primary localized orbital amyloidosis: a case report. Eur J Ophthalmol.

[5] Sharma VK, Agarwal M, Tyagi A, Sati A (2020). Primary localized conjunctival amyloidosis presenting corneal whorl-like opacity patterns. Cornea.

[6] Singh M, Rao KR, Das A (2018). Primary Orbital Amyloidosis. JAMA Ophthalmol.

[7] Caggiati A, Campanella A, Tenna S, Cogliandro A, Potenza C, Persichetti P (2010). Primary amyloidosis of the eyelid: a case report. In vivo (Athens, Greece).

[8] Al-Nuaimi D, Bhatt PR, Steeples L, Irion L, Bonshek R, Leatherbarrow B (2012). Amyloidosis of the orbit and adnexae. Orbit.

[9] Ali Z, Fernando B (2016). A rare case of amyloidosis of the eyelid and conjunctiva. Case Rep Ophthalmol Med.

[10] Sharma VA, Agarwal M, Tyagi A, Sati A (2020). Primary localized conjunctival amyloidosis presenting corneal whorl-like opacity patterns. Cornea.

[11] Kono S, Lee PAL, Kakizaki H, Takahashi Y (2021). Amyloidosis in the palpebral conjunctiva mimicking lymphoproliferative lesion. Case Rep Ophthalmol.

[12] Reynolds MM, Veverka KK, Gertz MA, Dispenzieri A, Zeldenrust SR, Leung N, Pulido JS (2018). Ocular manifestations of systemic amyloidosis. Retina (Philadelphia, Pa).

[13] Kang S, Dehabadi MH, Rose GE, Verity DH, Amin S, Das-Bhaumik R (2020). Ocular amyloid: adnexal and systemic involvement. Orbit.

[14] Jacoby S, Toft PB, Prause JU, Philip P, Heegaard S (2014). Nodular amyloidosis of all four eyelids: first presenting symptom of Waldenstrom macroglobulinaemia. Acta Ophthalmol.

[15] Choe JY, Kim N (2015). Secondary localized corneal amyloidosis caused by lower eyelid epiblepharon. Can J Ophthalmol Journal canadien d'ophtalmologie.

[16] Eneh AA, Farmer J, Kratky V (2016). Primary localized orbital amyloid: case report and literature review; 2004-2015. Can J Ophthalmol Journal canadien d'ophtalmologie.

[17] García de Oteyza G, de la Paz M (2016). Charoenrook de la Fuente V: Unilateral tarsal amyloidosis. Archivos de la Sociedad Espanola de Oftalmologia.

[18] Manaa Alkatan H, Al-Mohizea A, Alsuhaibani A (2017). A case of localized amyloidosis of the eyelid misdiagnosed as recurrent chalazion. Saudi J ophthalmol.

[19] Blandford AD, Yordi S, Kapoor S, Yeaney G, Cotta CV, Valent J, Perry JD, Singh AD (2018). Ocular adnexal amyloidosis: a mass spectrometric analysis. Am J Ophthalmol.

[20] Byers JT, Qing X, Lo C, French SW, Ji P (2018). Unilateral localized conjunctival amyloidosis in a patient with a history of contralateral orbit/eyelid lymphoma. Exp Mol Pathol.

[21] Hsiao PJ, Chang YC, Tsao YH, Wu KL, Kao YH, Chan JS, Wang CH, Lin YY, Chuu CP, Lin YS (2019). Ptosis and macroglossia in a woman with systemic light-chain amyloidosis. Clinica Chim Acta.

[22] Manjandavida FP, Chahar S (2019). Acquired progressive unilateral blepharoptosis. Indian J Ophthalmol.

[23] Medel Jiménez R, Sánchez España JC, Vasquez LM, Tapia Bahamondes A, Rondón M, Francesc T, Barroso EA (2019). Orbital and peri-orbital amyloidosis: a report of four cases. Orbit.

[24] Cui Y, Li B, Chen T, Zhao Y, Li DM (2016). Clinical and pathological features and surgical treatment of eyelid/conjunctival amyloidosis. (Zhonghua yan ke za zhi) Chin J Ophthalmol.

[25] Ma H, Lu C, Lai W (2018). Cutaneous amyloidosis presenting as bilateral nodular masses on the eyelids and cheeks. Ophthalmology.

